# A graph-based approach to multi-source heterogeneous information fusion in stock market

**DOI:** 10.1371/journal.pone.0272083

**Published:** 2022-08-11

**Authors:** Jun Wang, Xiaohan Li, Huading Jia, Tao Peng

**Affiliations:** 1 School of Management Science and Engineering, Southwestern University of Finance and Economics, Chengdu, China; 2 School of Computing and Artificial Intelligence, Southwestern University of Finance and Economics, Chengdu, China; 3 Management College, Ocean University of China, China Business Working Capital Management Research Center, Qingdao, China; DePaul University, UNITED STATES

## Abstract

The stock market is an important part of the capital market, and the research on the price fluctuation of the stock market has always been a hot topic for scholars. As a dynamic and complex system, the stock market is affected by various factors. However, with the development of information technology, information presents multisource and heterogeneous characteristics, and the transmission speed and mode of information have changed greatly. The explanation and influence of multi-source and heterogeneous information on stock market price fluctuations need further study. In this paper, a graph fusion and embedding method for multi-source heterogeneous information of Chinese stock market is established. Relational dimension information is introduced to realize the effective fusion of multi-source heterogeneous data information. A multi-attention graph neural network based on nodes and semantics is constructed to mine the implied semantics of fusion graph data and capture the influence of multi-source heterogeneous information on stock market price fluctuations. Experiments show that the proposed multi-source heterogeneous information fusion methods is superior to tensor or vector fusion method, and the constructed multi-attention diagram neural network has a better ability to explain stock market price fluctuations.

## 1. Introduction

As an important part of the overall financial market, the stock market has been widely studied by scholars and investors. Stock price trends are nonlinear and unstable time series, and scholars have extensively studied stock price fluctuations. In the past 30 years, China’s stock market, as the most rapidly developing emerging capital market, has become the focus of scholars’ research to prevent financial risks and obtain investment profits. In stock market research, scholars have proposed various theories to explain the volatility mechanism of the stock market, among which the most famous are the efficient market hypothesis and behavioral finance theory. The efficient market hypothesis [1] states that completely rational investors can engage in rapid and reasonable investment behavior given sufficient complete information. According to this theory, it is difficult for investors to achieve excess investment returns under the assumption of an efficient market. However, as financial behavior studies have intensified, scholars have found that investors are influenced by cultural and psychological factors, and financial behavior in the market is characterized by irrationality [2, 3]. In the research on stock market fluctuations, many factors have been found to affect stock market fluctuations, and different information can reflect the influence of different factors on stock market fluctuations. The interaction of various factors in the stock market has become a popular research direction [4, 5]. Currently, the amount of information is increasing at an astonishing speed due to the rapid development of information technology. Investors in the stock market are exposed to a substantial amount of information, which provides a good data basis for the research and analysis of stock market fluctuations. Trading data [6], such as opening price, closing price and trading volume, can be used to obtain the momentum [7], weight [8] and other derivative indicators for predicting and analyzing stock market price fluctuations. With the development of the internet and mobile internet technology, the impact of stock market news on stock market investors is becoming increasingly significant. Stock market news is all-inclusive, ranging from objective and neutral news reflecting stock market conditions and corporate business events to tendentious news reports that impact the short-term trend of the stock market [9, 10]. Researchers have proposed a large number of methods for stock market prediction based on sentiment analysis of stock market news [11, 12]. Simon et al. studied the quantity of news and concluded that a large amount of information would lead to poor attention [13]. Taking news headlines as the starting point, Chan studied investors’ sensitivity to news [14]. Wang et al. studied the impact of news reports on the stock market from two aspects: news media attention and emotion indicators [15]. Jue proposed an enterprise knowledge graph embedded system to expand enterprise-related news information. On this basis, the closed regression unit (GRU) model was used to predict stock price fluctuations by combining stock news sentiment and the quantitative characteristics of the considered stocks [16]. Furthermore, Nardo et al. verified that stock market information can be used for stock market prediction by studying a large amount of internet information, but their approach failed to achieve improved expected returns and was overly demanding for the construction of prediction models [17]. Analytical research on stock market price fluctuations has gradually developed from single indicators such as trading data and stock market news data to fused multi-indicator data. Multi-indicator data present multisource and heterogeneous characteristics. Thakkar et al. [18] classified stock market fusion techniques as into information fusion [19], feature fusion [20] and model fusion [21]. Information fusion is achieved mainly by extracting news information and fusing the data with stock market trading data to obtain comprehensive data for stock market forecasting. Feature fusion, on the other hand, uses algorithmic models to express homogenous data in different forms while achieving multidimensional feature extraction. In contrast, model fusion combines statistical models, artificial intelligence algorithmic models, deep learning models, etc., to form a composite model to analyze and predict stock market fluctuations. However, the current research suffers from several limitations, which are summarized as follows.

First, the feature extraction of news text is not sufficiently comprehensive. Information on emotional factors [11, 12], objective information [14] that responds to operational conditions and the means and amount of news coverage [13–15] contained in news texts can impact the stock market. The news text information in traditional multisource data fusion studies comprises features extracted from only a single perspective [18, 19]. Second, traditional multisource heterogeneous data fusion in the stock market is generally performed in a tensor or vector manner [22–25], and data redundancy is substantial given the current extremely large data volume and extremely fast information dissemination, which limits the efficiency of later data processing and analysis. When processing and analyzing multisource heterogeneous information, traditional econometric models cannot effectively capture and quantify the complex effects of nonlinear factors on asset volatility; furthermore, the black-box nature of machine learning algorithms has limitations in mining the implicit semantic information of complex financial data.

To address these challenges, we propose a graph fusion approach for multisource heterogeneous data and construct a graph neural network based on nodes and semantic attention mechanisms for predicting stock market price volatility trends. Specifically, we construct a complex network of multiple sources of heterogeneous information in the stock market to achieve heterogeneous data fusion of trading indicator data and stock market news text. A graphical neural network model based on multiple attention mechanisms is constructed to mine the interaction between multiple sources of heterogeneous information and the combined effect of information spillover on the stock market to predict stock market price fluctuations. The experimental results of stock market volatility prediction and strategy backtesting show that the proposed method achieves good results in stock market prediction.

In summary, the main innovative contributions of this study are as follows:

1. We efficiently quantify multisource heterogeneous information in the securities market, highlighting the financial characteristics of indicators. The multisource heterogeneous information is divided into heterogeneous nodes, and the information granularity is refined. Quantify the emotional features of news texts and set node features to integrate rational and irrational factors.
2. Innovatively introduce the relational dimension to build a complex network of time series. Based on effectively realizing the fusion of multisource heterogeneous information, it improves the efficiency of news mining in the securities market, solves the multiscale problem of financial time series, and interprets the correlation of time series.
3. We design a graph neural network with nodes and semantic attention as the core to capture the impact of information spillovers formed by interrelated multisource heterogeneous information on the price fluctuations of the securities market. Overcoming the limitations of traditional econometric models for nonlinear and complex financial data analysis, the deep learning model constructed in this paper is a very meaningful exploration of contemporary financial research.

## 2. Related work

### 2.1 Fusion technology

It has been proven in stock market research that stock price trend prediction is closely related to the characteristics of financial time series [26]. In fact, financial data have noisy, nonlinear, and random financial time characteristics, and there are many complex influencing factors [27]. However, Edwards R D et al. [28] proved that the trend of financial time series will recur, and the trend of individual special time series will appear very similar in the trend of future time series. Historical transaction data such as opening price, closing price, highest price, lowest price, and trading volume, can directly reflect changes in the financial market, and other technical indicators can be derived to assist in judging stock trends. AR, ARMA, ARIMA and optimization models use transaction time series data for linear analysis [29–31], while some models, such as RNN, CNN, and LSTM, process historical transaction data and derivative indicators into tensors for nonlinear analysis to predict stock market fluctuations [32]. Roondiwala [33] adopted a recurrent neural network (RNN) and long short-term memory (LSTM) for NIFTY50 prediction. With the deepening of research, text information is introduced into stock market quantification as an evaluation index, and text information is processed from the perspective of news event-driven and sentiment analysis. Atkins et al. [11] constructed a latent Dirichlet distribution model and used naive Bayes to predict the direction of stock market volatility through financial news. Wei et al. [12] constructed an aggregate news sentiment index for related companies, proving that sentiment levels in news reports can effectively act as a proxy to provide valuable support for portfolio decision making.

In recent years, scholars have studied and explored the fusion of multi-source heterogeneous data in the stock market. Zhang [34] proposed a new extended coupled hidden Markov model, which can effectively integrate news text and historical transaction data for stock prediction. In a later paper [19], events and user sentiments were extracted from online news and social media, and data fusion was achieved by coupling matrices and low-rank matrices decomposed by tensor framework. Kim [35] et al. proposed a hierarchical graph attention network method, which selectively aggregates information from different relationships, and the extracted relationship features initialize node features to realize information fusion for stock market forecasting. Li [36] obtained social emotions and professional opinions, used the tensor method to form the entire market information space composed of enterprise characteristics, and used a tensor learning algorithm information space to interact with stock trends. At present, multi-source heterogeneous data fusion methods based on the financial field convert data into vectors or tensors, but do not deeply explore the relationship between multi-source information, ignoring the dimensional correlation of time series.

### 2.2 Graph neural networks

Graph neural networks were developed to solve the deep learning problem of graph data. In just a few years, graph neural network technology has advanced considerably and has experienced wide application [37–39]. Bruna et al. [40] proposed graph convolutional neural networks in 2013 using a spectral space approach to define graph convolution. ChebNet [41] and GCN [42] define the weight matrix of nodes from a spatial perspective to reduce the spatiotemporal complexity and perform parameter optimization of the kernel function. Kim [35] et al. proposed a hierarchical attention network for stock market prediction using relational data. This method is used to predict the movements of individual stock prices and market indices by selectively aggregating information about different relationship types and adding this information to the representation of each company. Liu [43] et al. proposed a method to predict stock price fluctuations using a knowledge graph of relationships among listed companies using a closed-form regression cell model combined with related stock news sentiment, focal stock news sentiment, and number of focal stock news items. Matsunaga [44] investigated the effectiveness of cross-working between market forecasting and graphical neural networks by introducing company knowledge graphs into the forecasting model to mimic investors’ decision making. The validity in different markets and longer time spans was assessed using rolling window backtracking. Scholars have extensively explored the application of graph neural networks to stock market forecasting. The main role of graph data is to expand feature indicators based on relational data for stock market movement forecasting. To analyze and predict stock market fluctuations, the studies conducted thus far on the construction and analysis of complex networks of stock market information are in the initial stage. The impact of information spillover on stock market price volatility formed by the interconnection of multiple sources of heterogeneous information in the stock market requires further exploration.

In recent years, some efforts have been devoted to designing graph neural network learning for heterogeneous graphs. Wang [45] et al. proposed that the heterogeneous graph attention network mainly includes two types of attention mechanisms: node neighborhood and metapath. Fu [46] et al. took metapath nodes as the aggregation domain and proposed MAGNN (metapath aggregated graph neural network) to aggregate intrametapath information and intermetapath information. HetGNN [47] (heterogeneous graph neural network) first adopts a random walk strategy for neighborhood sampling and uses specialized Bi-LSTMs to fuse heterogeneous node features and neighborhood nodes. Hong [48] et al. designed HetSANN to learn different types of adjacent nodes and associated edges through a type-aware attention layer and directly encode information in heterogeneous graphs through a focused attention mechanism. On the basis of the transformer [49] architecture, an HGT (heterogeneous graph transformer) is built and applied to learn the features of different nodes and specific types of relations. However, most of the above methods follow the representation propagation mechanism of nodes and edges in spatially heterogeneous graphs, and the aggregation representation of nodes and edges in time series has not been further studied. To analyze and predict stock market fluctuations, the construction and analysis of the complex network of stock market information currently carried out are all in their infancy. The impact of information spillovers formed by interrelated multisource heterogeneous information in the stock market on stock market price fluctuations still needs to be further explored.

Based on previous research, this paper constructs a complex network based on multiple sources of heterogeneous information in the Chinese stock market, realizes effective data fusion, and introduces nodes and a semantic attention mechanism graph neural network to capture the interaction between multiple sources of heterogeneous information and the comprehensive impact of information spillover on the stock market. The proposed method is described in detail in the next section.

## 3. Model construction

### 3.1 Model introduction

**Fig 1:**
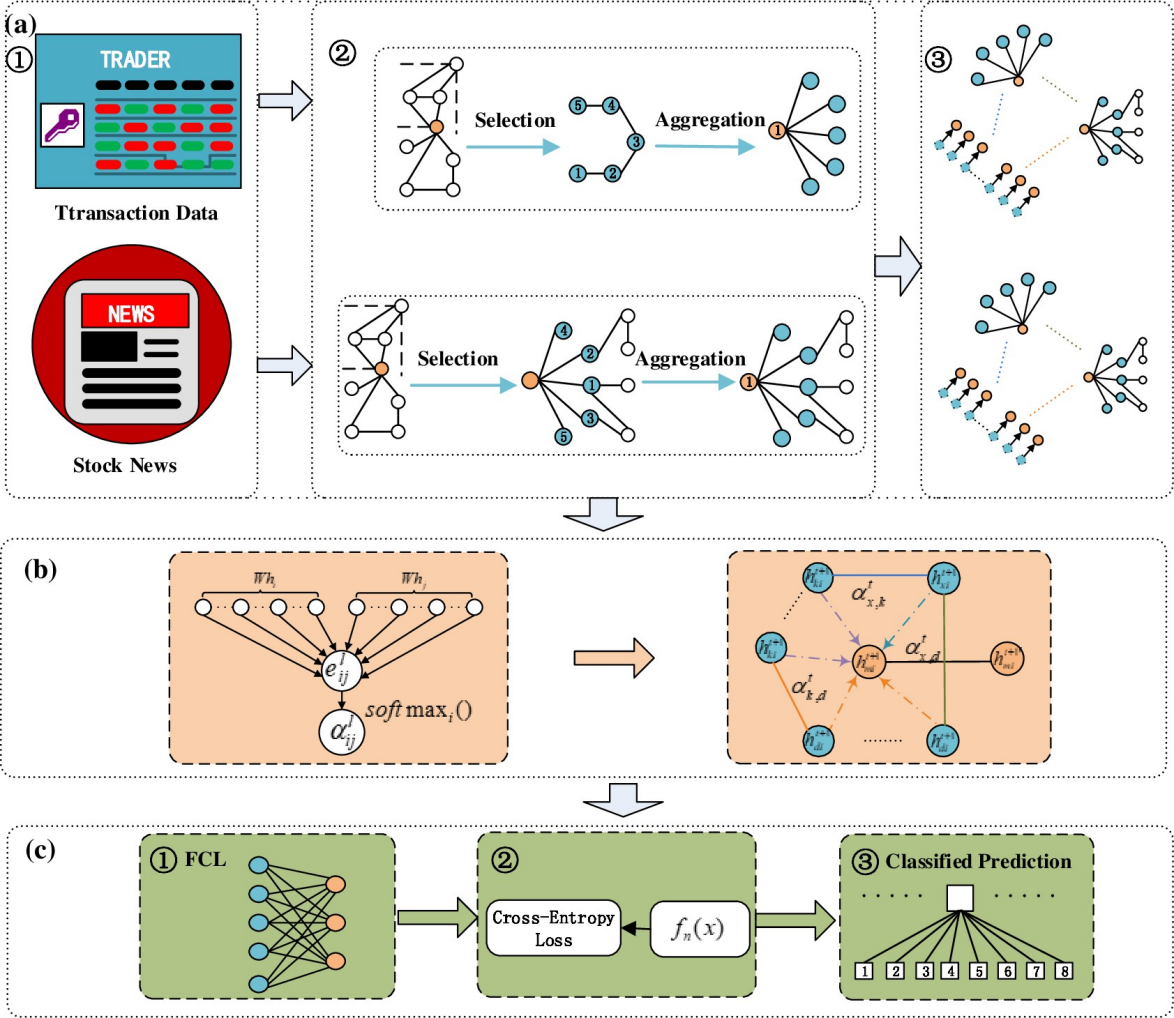
Overall framework of the model.

In this paper, a graph neural network model based on multiple attention mechanisms is proposed for stock market volatility prediction analysis. Fig 1 shows the overall framework of the model of the proposed method.

The model constructed in this paper defines stock market trading data and stock market news as different types of nodes and combines the characteristics of different types of nodes based on edges, weights of edges and information transfer direction using GRU for subgraph node aggregation, which is designed with a parallel structure to improve model efficiency. The aggregated vertices form the heterogeneous graph data, and the corresponding attention mechanisms of homogeneous and heterogeneous nodes are constructed by combining node semantics to accurately express the message overflow brought by information correlation between different semantics. Finally, the heterogeneous graph is classified to predict the stock market trend. In Fig 1, part (a) is the input stock market trading data indicators and stock market news used to construct the heterogeneous graph data; part (b) is the attention mechanism based on the heterogeneous graph data; and part (c) uses the fully connected layer and cross entropy function to classify and predict stock market fluctuations. Part (a) focuses on the data embedding and heterogeneous graph data construction process. (a) The main work in part ① is to use a web crawling engine for news data collection, integration with standard database news, and news text feature extraction via the BERT model. The text features and transaction indicator data are used as model input data to construct subgraph nodes and edge features. Part (a) ② performs subgraph construction for the two types of heterogeneous data input, combines various types of indicator features for node aggregation, sets up different types of edges between subgraphs, connects different subgraph aggregated nodes, sets up different types of edges between different types of nodes, and creates weight matrices for different types of edges. Part (a) ③ fuses the subgraphs, sets heterogeneous edges according to the node properties, and constructs a complex network based on multisource heterogeneous data of the stock market to accurately express the semantics of correlations between different indicators. Part (b) introduces nodes and semantic attention mechanisms to fully interpret the interaction effect and information spillover of different factors in the stock market according to the information meta-path of multisource heterogeneous data. Part (c) uses the fully connected layer and cross-entropy loss function to complete stock market trend prediction classification. Next, the key work of news text feature extraction, heterogeneous information data fusion, attention mechanism construction, and model construction will be elaborated.

### 3.2 Feature extraction

**Fig 2:**
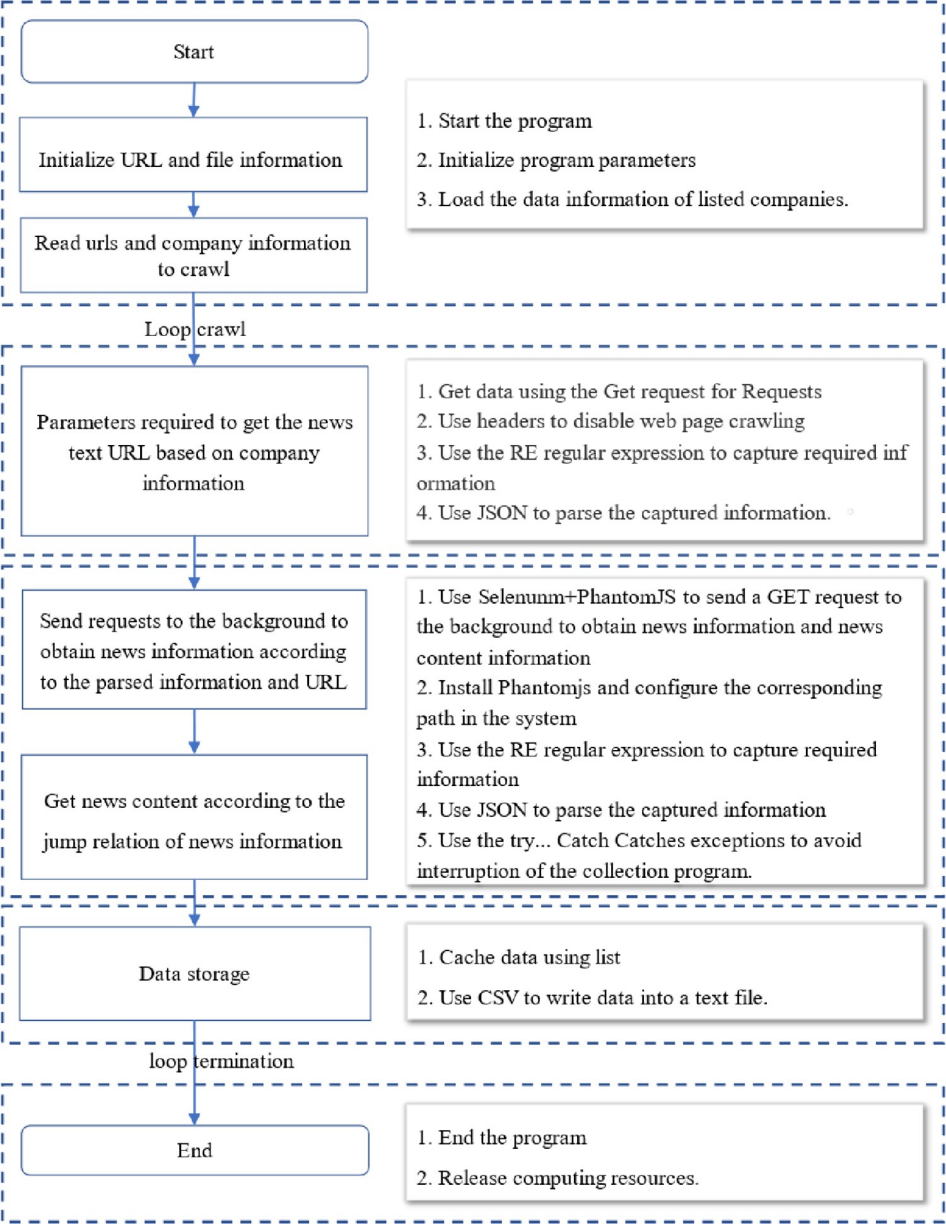
News text acquisition flowchart.

As stock market news text is unstructured data, the acquisition and quantitative representation is the key process of feature extraction of multisource heterogeneous data. In this paper, considerable effort is devoted to this process, including data acquisition and text quantification. To obtain more comprehensive news text data, this paper uses the Python language, builds a lightweight web crawling engine to complete the task, uses web crawler technology to simulate browser previews of the news of the listed companies to be studied, and obtains the required information through scripting programs. To improve the efficiency of information acquisition, the theme crawling method is chosen to crawl the text information directed to the news information on the specific web pages of aiqicha (https://aiqicha.baidu.com) and qichacha (https://www.qcc.com). The process of news text data collection on the internet is shown in Fig 2. In accordance with the experimental data settings used to establish a list of listed company names, the list data are loaded to initialize the URL, and the information of the company to be crawled is read according to the company information to obtain the parameters required for the news text, This process uses crawl data technology. Additionally, regular expressions are used to obtain key information via json parsing to finally identify the key information. The parsed information and URL are also used in the background to obtain news information The obtained news information is the news title text, and the news content and the news text information are obtained through regularized expressions and parsing. This process is simulated by PhantomJS, a webkit-based JavaScript API, which is directly driven by the program to achieve automatic web browsing, screenshotting, and other operations. This approach is used to crawl data from web news in this paper. Finally, the extracted news text is stored and cleaned, and the obtained news text information includes the news title, news content, release time, and source.

**Fig 3:**
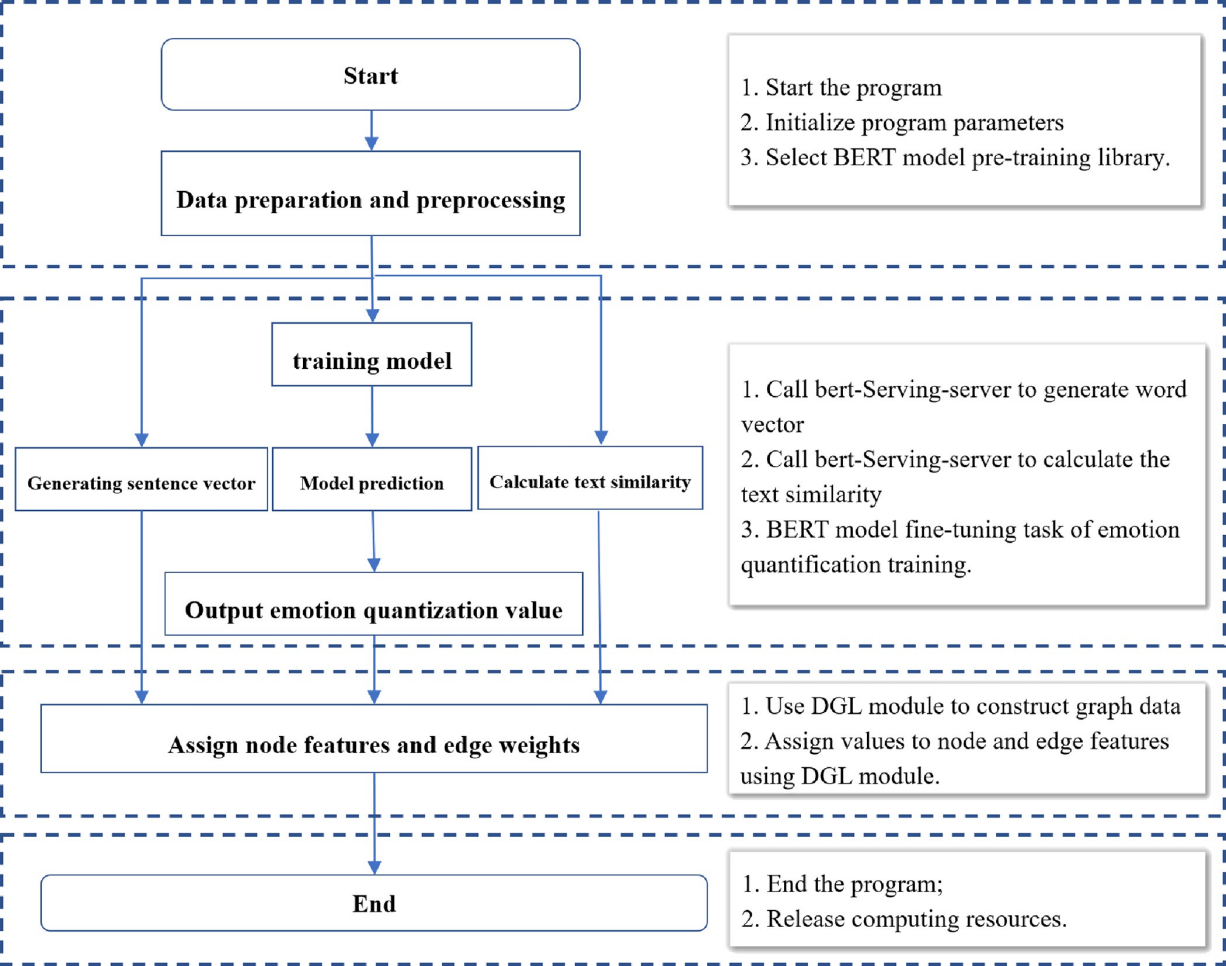
News text feature extraction flowchart.

The process of news text quantification and feature extraction is shown in Fig 3. The BERT model [50] pretraining library is used in the process of news text word vector and news headline similarity assessment with chinese_L-12_H-768_A-12.zip, and the two quantification processes remotely call the BERT service through the bert-serving-server using BertClient.endcode to obtain the sentence vectors. The news headline similarity is then calculated using the cosine angle. News text word vectors are used as content features of news subgraph nodes, and the similarity between news headline texts is assigned as the weights of the edges between corresponding news text nodes.

Investor sentiment is the focus of behavioral finance theory research, and news text in the stock market carries a large amount of sentiment information, which will directly affect investor sentiment and have a significant impact on stock market price fluctuations. The aim is to efficiently extract the sentiment information from the news text and enhance the fit between the sentiment contained in the news text and the stock market fluctuation; thus, a lightweight Chinese stock market sentiment corpus is constructed. First, quantitative sentiment classification is performed for stock market news headlines, and the stocks and their corresponding news headlines are labeled according to the T+1 trading day stock volatility Y results and labeled according to four categories of T trading day stock news headlines: Y≤-5%, -5%<Y<0, 0≤Y<5%, 5%≤Y, 1, 2, and 3. The data are divided into 3 parts, training, testing and validation, according to the ratio of 3:1:1. The processing classes are defined and registered, and the BERT model is trained. After subiterations, the final trained model is used to quantify news headline sentiment, and to avoid losses, the output is a probability (1*4 vector) for the classification of each news headline classified into the four classes. The output vector is used as the sentiment quantization feature of the news subgraph nodes in the next section.

### 3.3 Multisource heterogeneous data fusion

**Fig 4:**
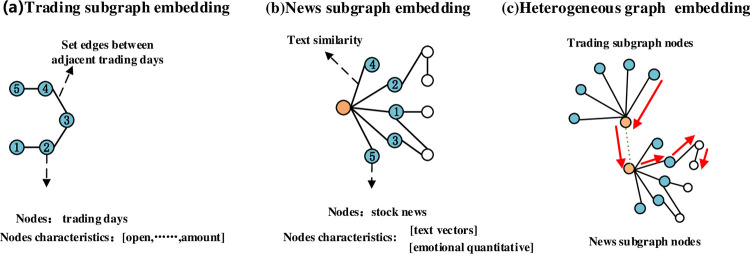
Graph fusion method ((a) trading subgraph embedding, (b) news subgraph embedding, and (c) heterogeneous graph embedding).

Fig 4 shows a schematic diagram of the subgraph data embedding method in Fig 1 part ①. In Fig 4(A), five trading days are selected as nodes to build the trading indicator subgraph. Based on the continuity of the time series of adjacent trading days, edges are set between trading day nodes. Six indicators, namely, the opening index, highest index, lowest index, closing index, volume, and turnover amount, are used as node characteristics. Fig 4(B) shows that the stock market news subgraph treats each trading day stock market news item as an indicator subgraph, with each news item as a subgraph node. The node features include two categories, the news text vector and sentiment quantization vector, as described in Section 3.2. The similarity between news texts is assigned as the weight of the edges to indicate the degree of connection between news texts. Fig 4(C) shows the heterogeneous network constructed based on two types of subgraphs with edges set in different types of nodes. The types of edges in the complex network have three main types: edges between transaction indicator nodes, edges between news text nodes and edges connected between transaction indicator nodes and news text nodes.

### 3.4 Attention mechanism construction

The heterogeneous graph G = (V, E) consists of two types of indicator node sets and connection sets. The meta-paths are shown as red arrows in Fig 4 are sequences of complex network graph data traversing a set of heterogeneous nodes constructed in this paper, and the nodes through which each meta-path passes are defined as neighborhood nodes.

**Fig 5:**
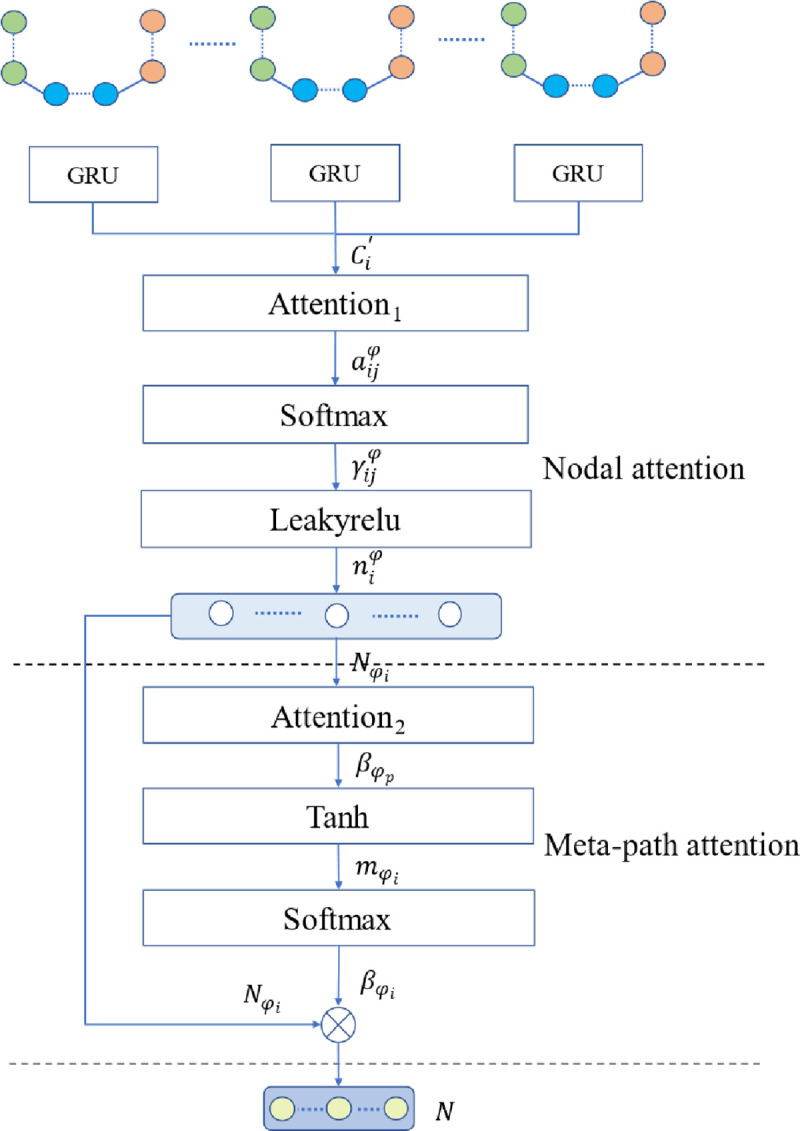
Overall flow chart of the attention mechanism.

In stock market volatility prediction, each type of indicator node presents different importance on the meta-path, and the attention mechanism for the nodes is based on the heterogeneous node neighborhoods constituted by the path. The attention mechanism construction process is shown in Fig 5. To clearly explain the model constructed in this paper, Table 1 shows the names and meanings of the explanatory variables.

**Table 1:** Explain variable names and meanings.

Variable name	The variable meanings
*C*_*i*_	The original node feature, which includes the news text node sentence vector and the transaction data node transaction data vector.
$C_{i}^{\prime },C_{j}^{\prime }$	To map the transformed node feature space through GRU, it includes news text nodes and transaction data nodes, unifies the vector dimension, and forms a feature space, which effectively improves the processing efficiency of heterogeneous nodes.
*φ*	The meta-path is the path that traverses different types of nodes at the same time t as shown in Fig 4(C), and its main function is to determine the field of information overflow process nodes.
*W*_1_, *W*_2_	*W*_1_ is the feature weight matrix converted from the heterogeneous node feature map, indicating the importance of each node *W*_2_ is the feature weight matrix of the aggregated nodes according to the meta-path, indicating the importance of the aggregated nodes after the node attention processing.
$\gamma_{ij}^{\varphi }$	The weight coefficient is used to express the importance of each node in the neighborhood with the meta-path as the neighborhood.
$n_{i}^{\varphi }$, $N_{\varphi_{i}}$	$n_{i}^{\varphi }$ is the aggregated vertex feature, which reflects the node feature and its importance in the domain based on the meta−path. $N_{\varphi_{i}}$ represents a node set composed of aggregated nodes $n_{i}^{\varphi }$.
$m_{\varphi_{i}},\beta_{\varphi_{i}}$	$m_{\varphi_{i}}$ is the weight value of each meta−path, indicating the importance of each meta−path; $\beta_{\varphi_{i}}$ is the weight coefficient of each meta-path, which is obtained by normalizing the weight value, and the weight coefficient indicates the importance of the meta-path in the interpretation of the price fluctuation of the securities market.

However, for the two different types of nodes with heterogeneous node features, the feature space of heterogeneous nodes is first converted. The node feature conversion is performed using GRU, which is shadowed to a unified feature *C*_*i*_ space for the original node $C_{i}^{\prime }$ features and for the node feature space after conversion by mapping.


$$C_{i}^{\prime }=GRU(C_{i})$$ (1)

The attention mechanism for nodes *Attention*_1_ is used mainly for identification under a *φ* meta-path, i.e., under a neighborhood. The importance of different types of nodes to each other is expressed by the weights formed by the attention mechanism.

$$a_{ij}^{\varphi }=Attention_{1}(C_{i}^{\prime },C_{j}^{\prime },\varphi )$$ (2)

$a_{ij}^{\varphi }$ is asymmetric, i.e., two nodes may differ from each other, i.e., there is directionality on the meta-path. The importance weights between two nodes are calculated as a more accurate expression of the weight *φ* coefficients under the $\gamma_{ij}^{\varphi }$ path. *softmax* is introduced to perform the normalization operation. The activation function in this paper is LeakyReLU because the previous operations are linear. The function increases nonlinear factors, thereby improving the efficiency of mining useful information from complex networks. Another role is to limit the data expansion and alleviate the memory overflow phenomenon when actually processing data. ‖ denotes the feature connection operation.


$$\gamma_{ij}^{\varphi }=soft\max_{j}(a_{ij}^{\varphi })=\frac{\exp(\sigma (W_{1}[C_{i}^{\prime }\|C_{j}^{\prime }]))}{\sum_{k\in \varphi }\exp(\sigma (W_{1}[C_{i}^{\prime }\|C_{k}^{\prime }]))}$$ (3)

Based on meta-path *φ*, the vertex nodes $n_{i}^{\varphi }$ are aggregated from the weight coefficients $\gamma_{ij}^{\varphi }$ with the corresponding meta-path (neighborhood) nodes. Eq (4) reflects the node aggregation process of node i features, neighborhood *j*∈*φ* node features $C_{j}^{\prime }$ and the importance $\gamma_{ij}^{\varphi }$ (weight coefficients) among meta-path nodes.


$$n_{i}^{\varphi }=\sigma (\sum_{j\in \varphi }\gamma_{ij}^{\varphi }.C_{j}^{\prime })$$ (4)

In view of the small-world and law-distribution characteristics of the complex network of the stock market constructed in this paper, to stabilize the training characteristics of the model and reduce the impact of excessive variance of the graph data, the model uses multiheaded attention and repeats the attention mechanism construction operation K times, as shown in Eq (5). Finally, the embedded nodes $n_{i}^{\varphi }$ are obtained via node-level attention.


$$n_{i}^{\varphi }=\overset{K}{\underset{k=1}{∥}}\sigma (\sum_{j\in \varphi }\gamma_{ij}^{\varphi }.C_{j}^{\prime })$$ (5)

In the node embedding process constructed above based on the importance of different types of nodes to each other, there is another problem to solve, i.e., the stock market complex network graph data construction process, in which the nodes between different types are connected with edges. The premise of the edge setting assumes extensive connections and interactions between different types of indicators that jointly impact price fluctuations in the stock market. Therefore, the edge setting traverses the whole complex network, and although the edge types are limited and the meta-paths are short, a large number of redundant edges remain, which seriously affects the model training and prediction performance. To further explore the importance and implied information of different meta-paths, the embedded node group formed by node-level attention is used to construct each meta-path weight based on the attention mechanism of different *Attention*_2_ meta-paths. As shown in Eq (6), the input is the aggregated node group $\left(N_{\varphi_{1}},N_{\varphi_{2}}\cdots N_{\varphi_{p}}\right)$ based on meta-paths, and the output is the meta-path attention weights $\left(\beta_{\varphi_{1}},\beta_{\varphi_{2}}\cdots \beta_{\varphi_{p}}\right)$.


$$\left(\beta_{\varphi_{1}},\beta_{\varphi_{2}}\cdots \beta_{\varphi_{p}})=Attention_{2}(N_{\varphi_{1}},N_{\varphi_{2}}\cdots N_{\varphi_{p}}\right)$$ (6)

The nodes $n_{i}^{\varphi }$ after aggregation according to the meta-path group are nonlinearly *tanh* transformed and calculated as the inner product of the meta-path weight vector *a*^*T*^, as shown in Eq (7). The importance $m_{\varphi_{i}}$ of each meta-path is also calculated. *W*_2_ is the feature weight matrix of the aggregated node, *h* is the bias, and $\frac{1}{\left|V\right|}$ indicates the normalization of the meta-path importance.


$$m_{\varphi_{i}}=\frac{1}{\left|V\right|}\sum_{i\in V}a^{T}•\tanh(W_{2}•n_{i}^{\varphi }+h)$$ (7)

Eq (8), based on the importance $m_{\varphi_{i}}$ of the meta-paths obtained, uses *softmax* normalization to obtain the weight coefficient $\beta_{\varphi_{i}}$ of each meta-path. The weight coefficient indicates the importance of the meta-path for the complex network classification prediction of the stock market.


$$\beta_{\varphi_{i}}=\frac{\exp(m_{\varphi_{i}})}{\sum_{i=1}^{P}\exp(m_{\varphi_{i}})}$$ (8)

Different meta-path weight coefficients $\beta_{\varphi_{i}}$ and node-level attention mechanisms $N_{\varphi_{i}}$ are combined to obtain the final aggregated nodes $N_{\varphi_{i}}$, the final aggregated nodes are aggregated according to the meta-paths *φ*_*i*_, and the final aggregated nodes *N* fully exploit the importance information embedded in the node and semantic layer by means of two types of attention mechanisms on the basis of containing the traditional node features.


$$N=\sum_{i=1}^{P}\beta_{\varphi_{i}}N_{{}_{\varphi_{i}}}$$ (9)

In this paper, the stock market complex network graph data are used to complete the classification prediction of changes in stock market prices, so the cross entropy function shown in Eq (10) is used to minimize the gap between the distribution of predicted stock market rising/falling situations and the actual labels to complete the training and prediction of the model. C is a parameter of the classification part, *y*_*L*_ represents the data set that indicates the labels of the stock market complex network graph data, and *Y*^*l*^ and *N*^*l*^ represent the real and predicted labels, respectively.


$$L=-\sum_{l\in y_{L}}Y^{l}ln(C•N^{l})$$ (10)

## 4. Experimental simulation and results analysis

### 4.1 Data set introduction

**Fig 6:**
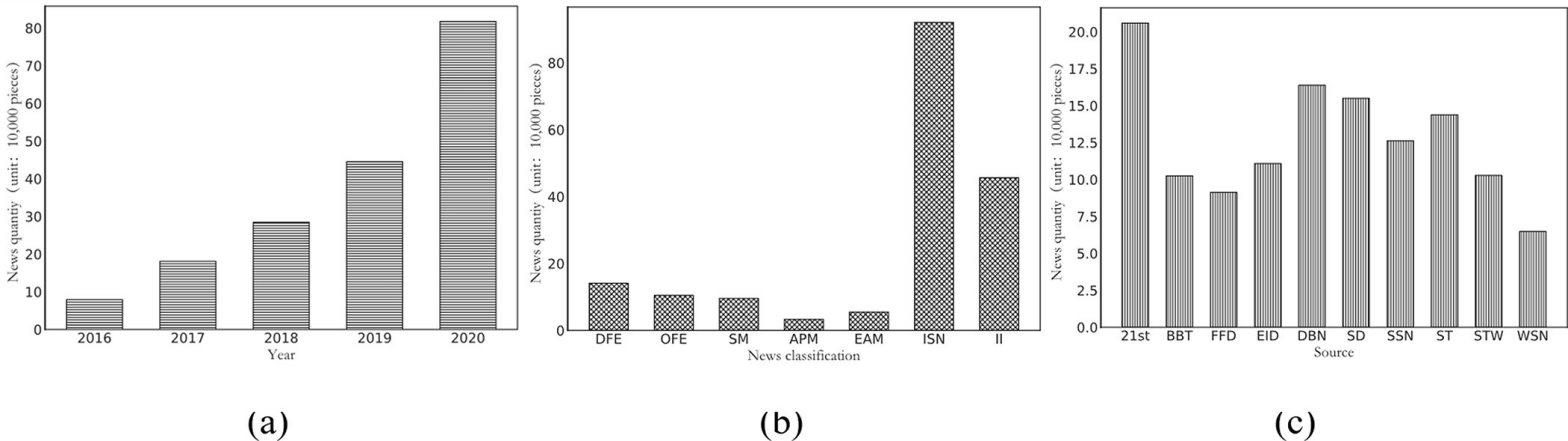
Distribution of news((a) the distribution of the number of news texts per year (b) the classification of news text data, (c) the distribution of news sources).

The data used in this paper are obtained from the CMSAR database (China Stock Market & Accounting Research Database) of CSI 300 constituent stocks from January 1, 2016, to December 31, 2020. The stock news data used in this paper are from the CMSAR database and internet crawling, and the internet crawling was performed via aiqicha (https://aiqicha.baidu.com) and qichacha (https://www.qcc.com). The total number of texts between January 1, 2016 and December 31, 2020, for the 300 stocks is 1807207 (2016: 79091; 2017: 180868; 2018: 284679; 2019: 445356; 2020: 817213). The database news texts were classified as domestic finance, overseas finance, securities market, Asia-Pacific stock market, European and American market, individual stock news, and industry information (from Xinhua, 21st Century Business Herald, Economic Observer, Yangzi Evening News, and other media, the data is available at the following link. https://figshare.com/s/7f924a6df9b4a345a3f0). Table 2 provides an overall description of the distribution of transaction data and news texts, and shows the number of days of trading and the number of news texts. Table 3 is the statistical description of stock market transaction data, Fig 6 is the distribution of news text data, and Fig 6(A) is the distribution of the number of news texts per year. The development of technology shows a trend of increasing year by year. Fig 1(B) shows the classification of news text data. The classification is based on the news classification of the CMSAR database (China Stock Market & Accounting Research Database). It is divided into 7 categories: domestic finance and economics (DFE), overseas finance and economics (OFE), securities market (SM), Asia-Pacific market (APM), European and American markets (EAM), individual stock news (ISN), and industry information (II). Fig 6(C) shows the distribution of news sources, with the top 10 news sources: 21st Century Business Herald (21st), Daily Business News (DBN), Securities Daily (SD), Securities Times (ST), Shanghai Securities News (SSN), Economic Information Daily (EID), Securities Times Website (STW), Beijing Business Today (BBT), First Financial Daily (FFD), and Wall Street News (WSN). According to the classification and sources of news texts in Fig 6(B) and 6(C), it can be seen that the news text data, as the core experimental data of this paper, have a reasonable distribution, and the news coverage is relatively comprehensive.

**Table 2:** Experimental data distribution.

Year	2016		2017		2018		2019		2020	
Industry	Days	News	Days	News	Days	News	Days	News	Days	News
**Financial**	11576	13088	12591	33168	14576	59234	13889	84866	15224	170957
**Public utilities**	13584	15358	14071	37067	11903	48373	11691	71436	11172	125456
**Real estate**	5198	5877	6660	17544	6728	27341	5611	34285	5811	65254
**Comprehensive**	972	1099	1159	3053	616	2503	488	2982	729	8186
**Industrial**	36321	41064	31275	82386	33880	137682	39011	238369	37893	425519
**Business**	2304	2605	2904	7650	2349	9546	2196	13418	1945	21841

**Table 3:** Transaction data distribution.

Indicators	Open	High	Low	Close	Volume	Turnover
**Mean**	23.84	24.23	23.49	23.87	5.13e+07	1.03e+03
**Std**	62.70	63.54	61.95	62.81	1.10e+08	1.45e+09
**Min**	1.04	1.07	1.04	1.05	4.90e+05	2.74e+06
**25%**	6.69	6.78	6.60	6.69	1.04e+07	2.21e+08
**50%**	11.8	12.01	11.63	11.82	2.29e+07	4.94e+08
**70%**	24.53	24.97	24.17	24.56	5.57e+07	1.18e+09
**Max**	1941.00	1998.98	1939.00	1998.00	2.17e+09	1.63e+10

**Fig 7:**
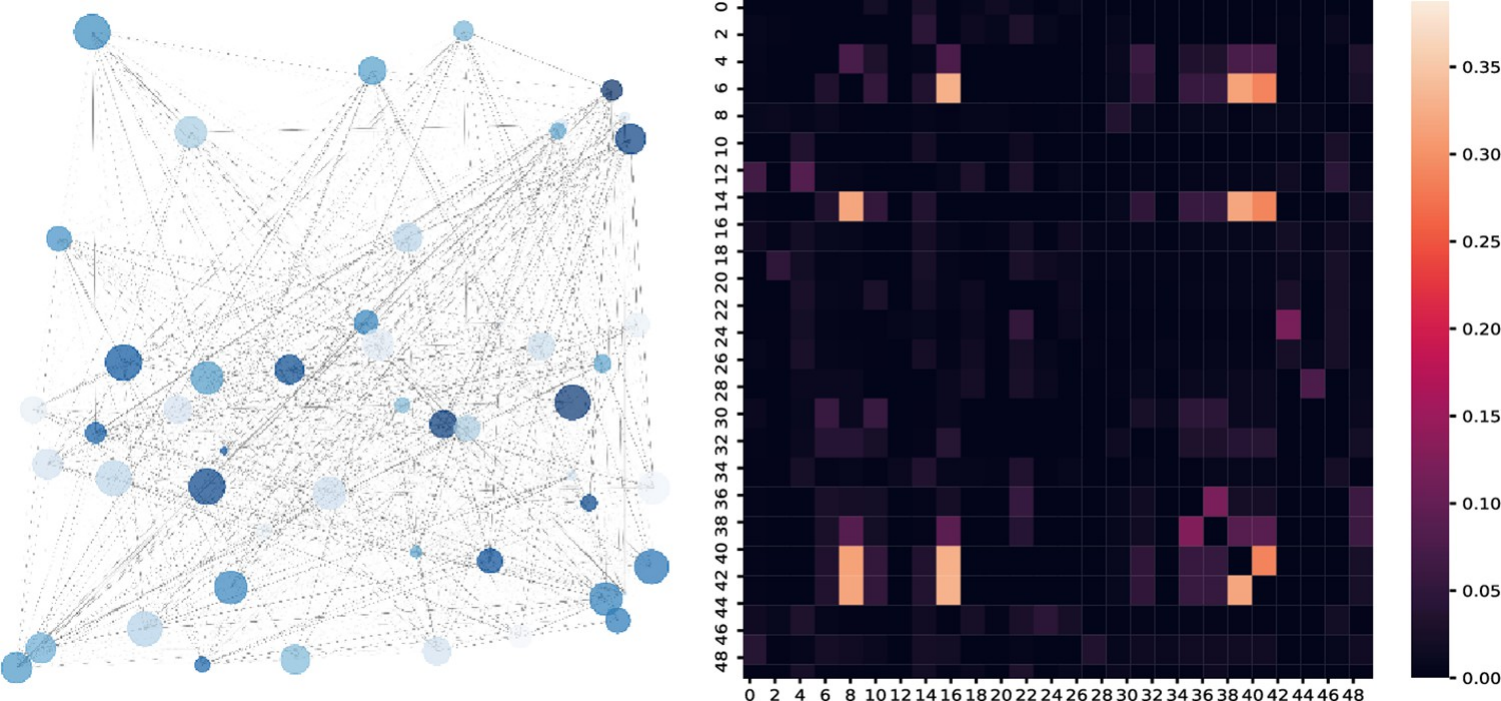
Example stock market news graph data.

The graph data are constructed using dgl, and the corresponding convolution operations are performed. The gensim library is used to convert the news text into 200-dimensional word vectors as node features. The parameters of word2vec are set as size = 20, window = 7, min_count = 0, sg = 1, alpha = 0.15, iter = 10, and batch_words = 10000, and the other parameters are set to the default values. The similarity model calculates the similarity between news texts as the weights of the news subgraph edges. The trading data indicator subgraph selects the five working days before the forecast, uses the six indicators of the opening index, highest index, lowest index, closing index, volume, and turnover amount of each trading day as the node features after aggregation, and splices them with the aggregated vertex features by means of a concatenate operation. The left side of Fig 6 shows the graph data of 50 stock market news nodes on random trading days. The depth of the edges in the graph is proportional to the news text similarity, and the node size is proportional to the node degree. The right side of Fig 7 shows the similarity of 50 random news nodes, the horizontal and vertical axes are 50 news nodes, and the color in the figure identifies the similarity between the news.

### 4.2 Feature extraction results

**Fig 8:**
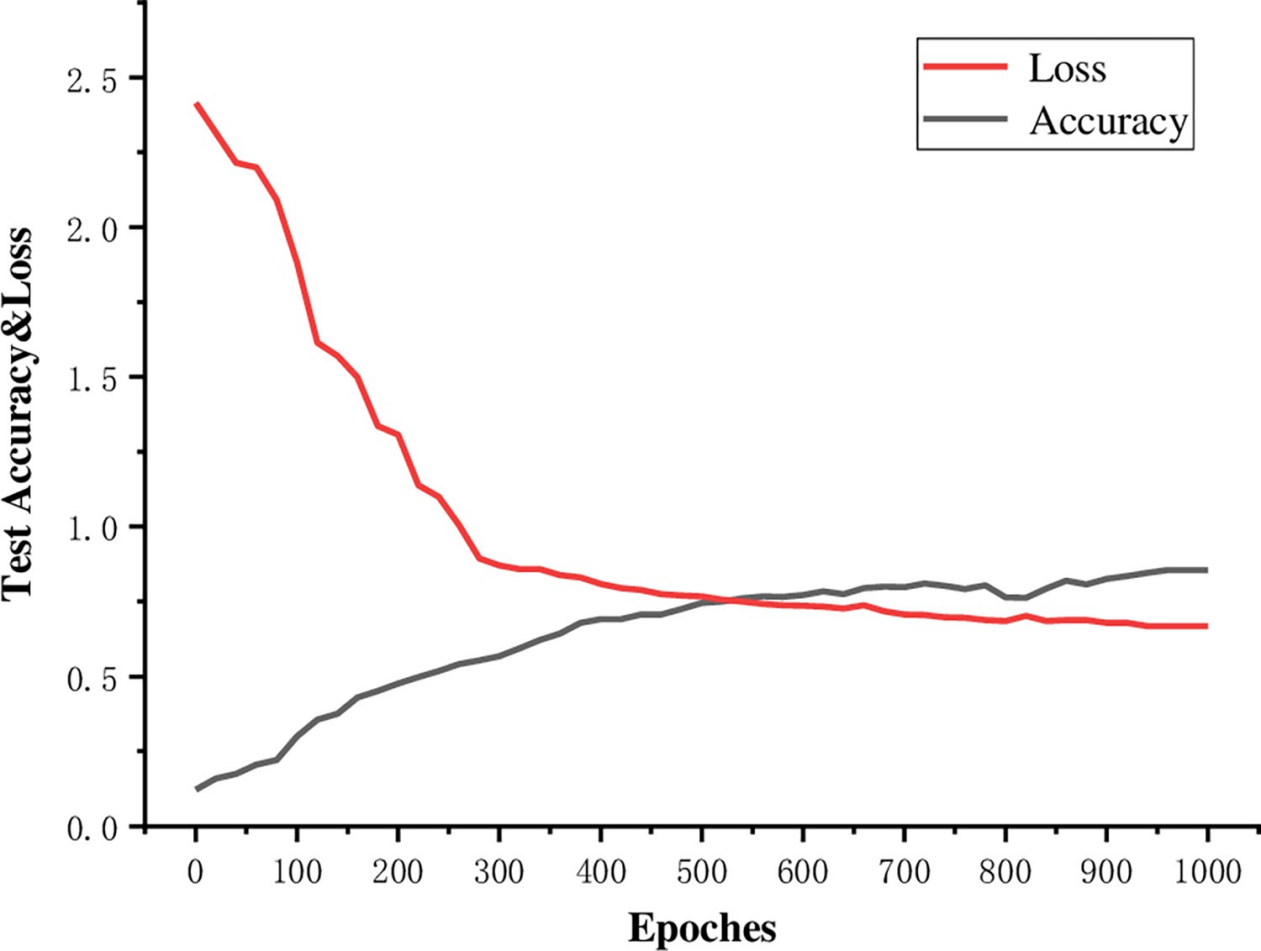
BERT model training process.

Because news text sentiment quantification is the key to feature extraction, this paper uses the BERT model for news text sentiment quantification feature extraction. The BERT model uses a transformer bidirectional encoder and pretraining to effectively improve the model performance. To ensure the objectivity of subsequent experiments, this paper uses data from 2016 to 2019 for training, validation and prediction. The data were divided according to a 3:1:1 ratio. The total number of training samples is 989994, and the classification distribution is [593998, 197998, 197998]. The model parameters are set as follows. require_improvement = 10000, i.e., the training process is terminated if the model performance is not improved after 1000 batches. num_classes = len(class_list), i.e., the number of labels is set to 4 classes. num_epoch = 1000: the number of epochs is set to 1000 to improve the training efficiency. batch_size = 10, and learning_rate = 5e-5. The training process parameters are set as follows: TOTAL_ITERATIONS = 1000 (the total number of iterations is set to 1000), VALID_ITERATION = 50 (validation is performed every 50 rounds), DELAY_NUM = 100 (if the prediction performance is not improved after 100 iterations, then training is terminated), USE_L2 = True (a regularization method is applied), and L2_LAMBDA = 0.04 (the regularization parameter is set to 0.04). Fig 8 Appearance the BERT model process training. With the increase of training times, the loss value and the accuracy rate tend to be smooth, and the model has converged.

The BERT model achieves an accuracy of 85.59% after training, and all feature extraction training and testing processes are conducted using data from 2016 to 2019 to ensure the objectivity of the subsequent experimental results. The test set used to validate the data fusion and strategy backtesting experiments was not within the scope of the feature extraction experiments. The 2020 news text is only output as the result of the feature extraction method, and the feature vector is the sentiment quantification of the news subgraph nodes.

### 4.3 Data fusion and classification prediction performance

A confusion matrix of the results is shown in Table 4, and rising and falling stock market closing prices are used as the classification prediction results. If the prediction result and the actual result are both rising, it is a true positive (TP). If the predicted result is rising and the true result is falling, it is a false positive (FP). A false negative (FN) occurs if the predicted result is falling and the true result is rising. If the predicted result is falling and the true result is also falling, the result is a true negative (TN).

**Table 4:** Confusion matrix.

Confusion matrix		Predicted value	
		Up	Down
True Value	Up	TP	FN
	Down	FP	TN

The performance of the proposed stock market data fusion analysis forecasting method is measured according to references [19, 23–25] based on the accuracy (accuracy), F1 value and Mathews correlation coefficient (MCC). The accuracy, F1 value and Mathews correlation coefficient are shown in Eqs (11)–(13).


$$Accuracy=\frac{TP+TN}{TP+FN+FP+TN}$$ (11)

$$F1=\frac{2Precision*Recall}{Precision+Recall}$$ (12)

$$MCC=\frac{TP*TN-FP*FN}{\sqrt{\left(TP+FP)(TP+FN)(TN+FP)(TN+FN\right)}}$$ (13)

To further verify the superiority of the constructed model when applied to massive news text information, this paper introduces the multisource metric methods SVM, RandomForest, MKKM [23], and TeSIA [24] for model comparison. The comparison methods do not consider the relational dimensions between news data but only simple vector data splicing, and the model parameters introduced for comparison are shown in Table 5.

**Table 5:** Model hyperparameter settings.

Method	Parameters
SVM	C = 0.8; kernel = linear; max_iter = 1000
RandomForest	Max_feature = none; min_samples_split = 10; n_estimators = 3
MKKM	View = 3; kernel = RBF; gamma = 1/3; k = 2
TeSIA	Tensor_order = 3; tensor_size(i = 5, j = 1, k = 10); Max_iter = 5000

**Fig 9:**
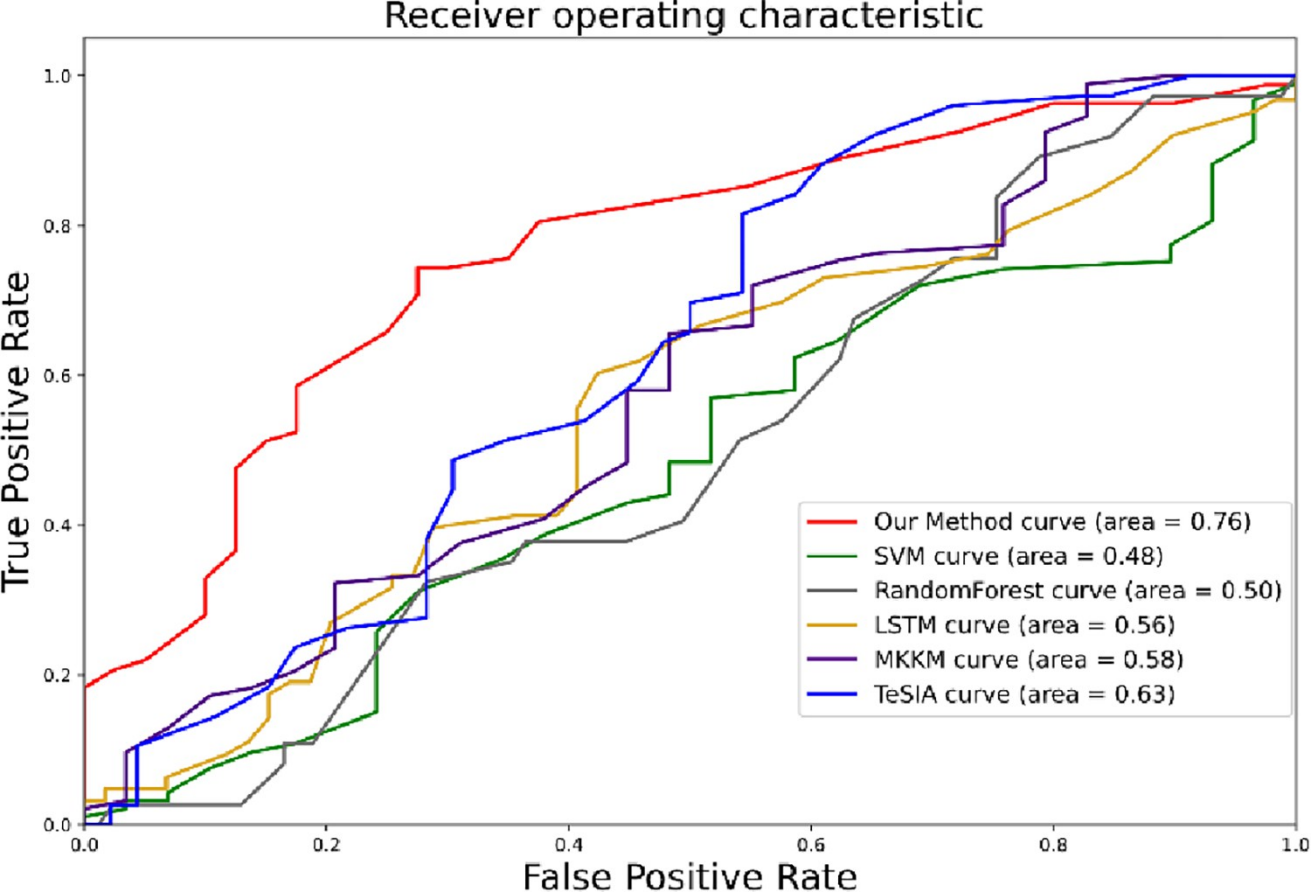
ROC/AUC plot for predicting the classification of 300 CSI constituents.

The experimental results in Table 6 and Fig 9 show that in terms of data fusion, SVM adopts the method of fusing multi-source heterogeneous information in the form of vectors and tensors, and the accuracy rates are 47.47% and 46.23%, respectively, and the accuracy rates are basically maintained near-random probability. RandomForest is slightly better than the SVM method, based on the traditional vector and tensor fusion method, and it is always close to random probability. The prediction accuracy of the vector embedding method is slightly better than that of the tensor method. The vector method has weight data sharing between features, and the tensor method lacks the weight sharing of some subindicators. MKKM and TeSIA take into account the relationship between the three types of indicators, the prediction performance is effectively improved, and the accuracy, F1 value, and Matthews correlation coefficient are better than those of the RF and SVM methods. It verifies the importance of the implicit semantics of the relationship between multi-source heterogeneous information for financial time series forecasting. The method adopted in this paper reasonably represents the relationship information of multi-source heterogeneous indicators via graph fusion and analyzes the relationship information according to different types of information. Quantization fully improves the fusion representation efficiency of multi-source heterogeneous information. It provides the possibility to mine the effective information of securities market news and solve the multi-scale problem of financial time series. In terms of fusion information data processing, multi-source and isomerized data have a large amount of information, and there is a problem of information overload. Although MKKM, TeSIA and LSTM methods all use deep learning technology, in the face of a large amount of information, multi-source,they are multisource and heterogeneous. Traditional deep learning technology has limitations in key information location and information mining. Based on the theoretical basis of information spillover in the financial market, this paper designs an attention mechanism graph neural network to fully simulate the influence of information flow and diffusion on the price fluctuation of the securities market. It effectively improves the efficiency of information mining, solves the problem of information overload, and effectively improves the efficiency and accuracy of forecasting price fluctuations in the securities market. The method proposed in this paper is based on the advantages; it is the best prediction performance model among the above two related prediction methods, and the accuracy rate, F1 value and Matthew coefficient (MCC) are 69.36%, 0.781, and 0.3, respectively. The three types of indicators improved by 6.23%, 0.104, and 0.011 compared with the previous 17 models. In comparison, the superior results proposed in 8 indicate that the method proposed in this paper has obvious advantages in prediction accuracy and stability compared with the SVM, RandomForest, MKKM, LSTM, and TeSIA methods.

**Table 6:** Summary of CSI 300 constituent stock strategy returns.

Stock	Benchmark Return	Strategy Return	Alpha	Beta	Sharpe Ratio	Sortino Ratio	Information Ratio	Maximum Drawdown
CSI 300	-14.37%	2.35%	0.36	1.312	0.681	1.016	1.628	27.17%

### 4.4 Strategy backtesting

**Fig 10:**
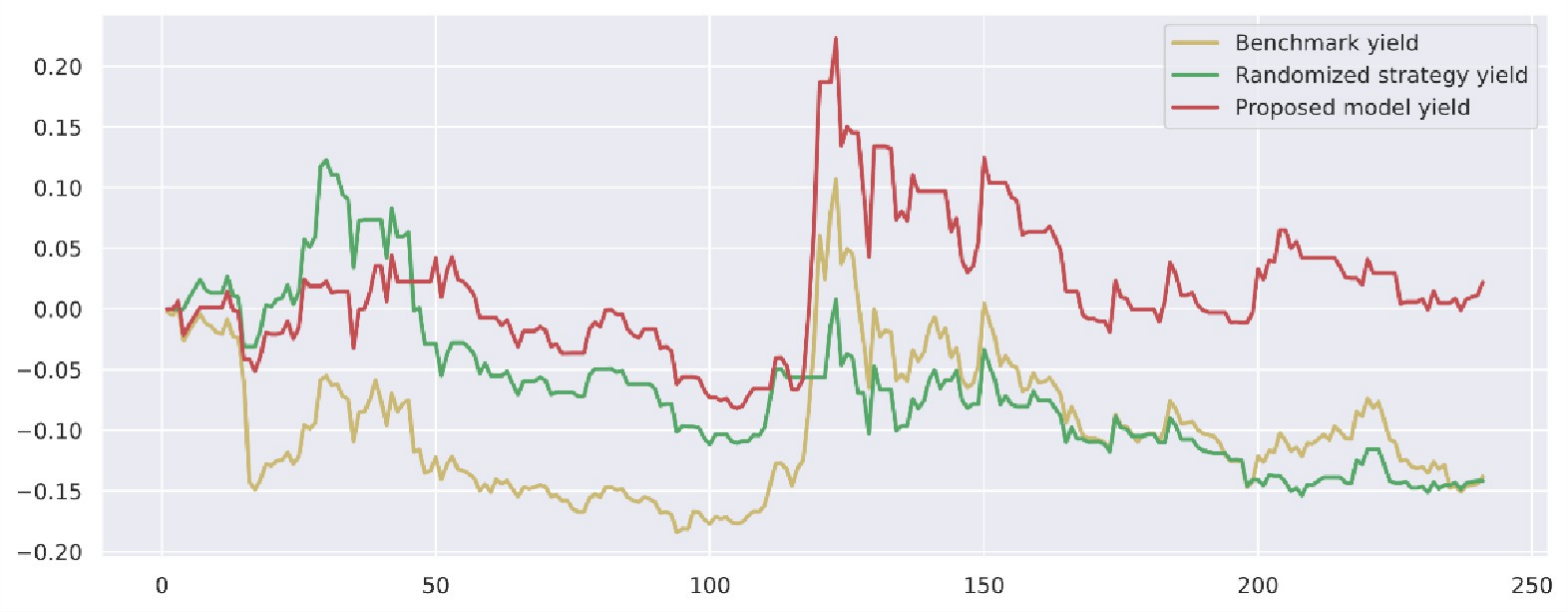
CSI 300 constituent stock strategy returns.

**Fig 11:**
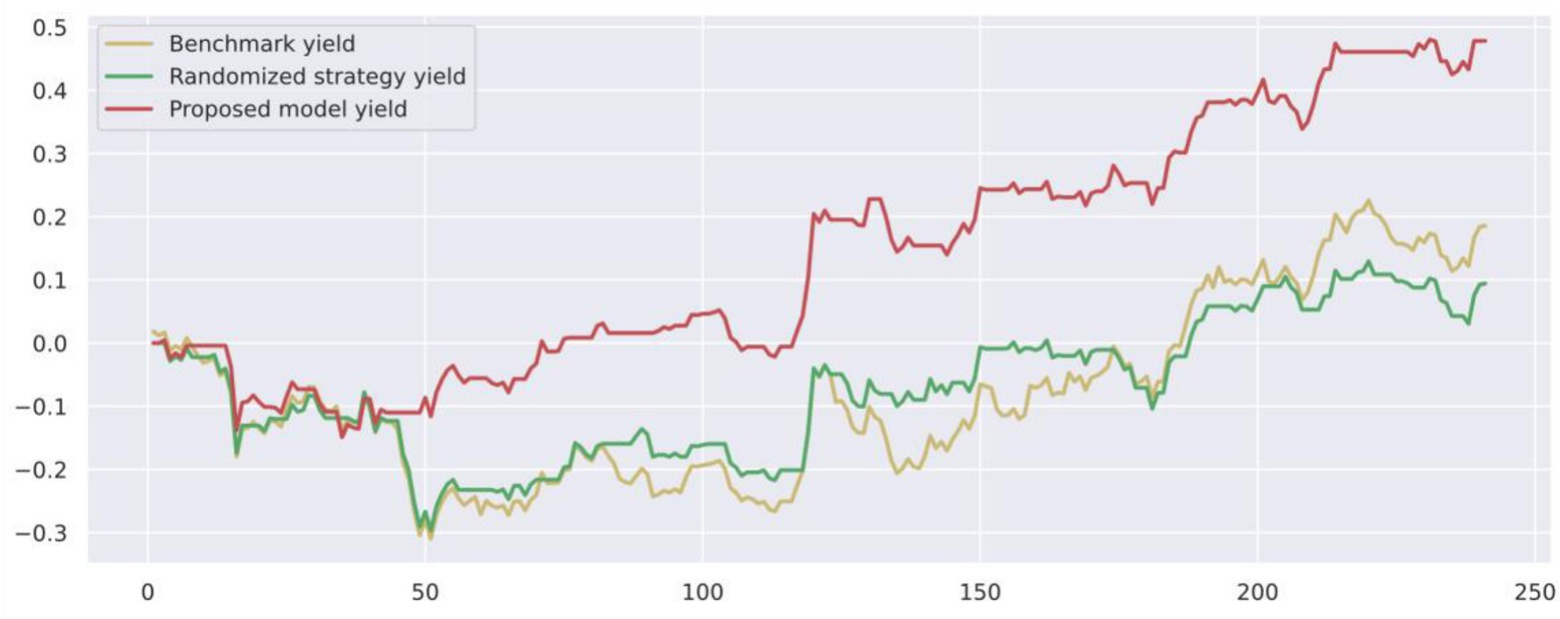
Ping An Bank stock strategy returns.

**Fig 12:**
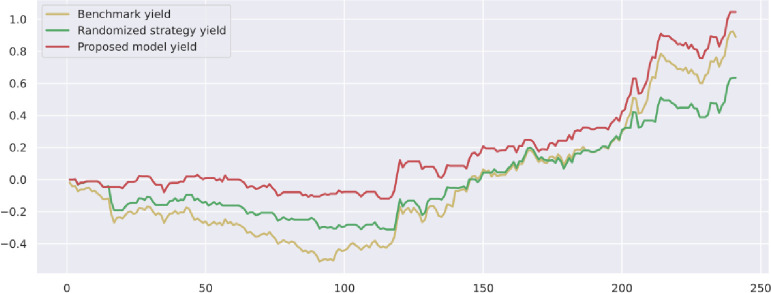
China Ocean holdings stock strategy returns.

**Fig 13:**
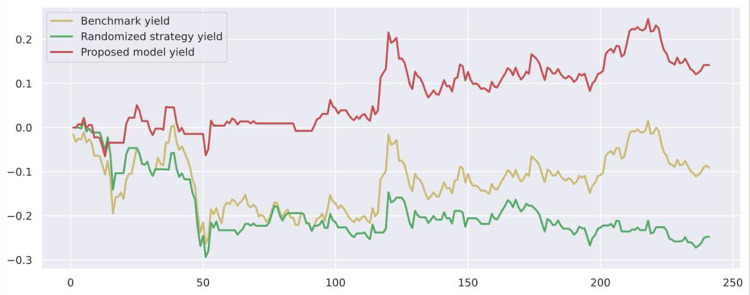
Vanke A stock strategy returns.

**Fig 14:**
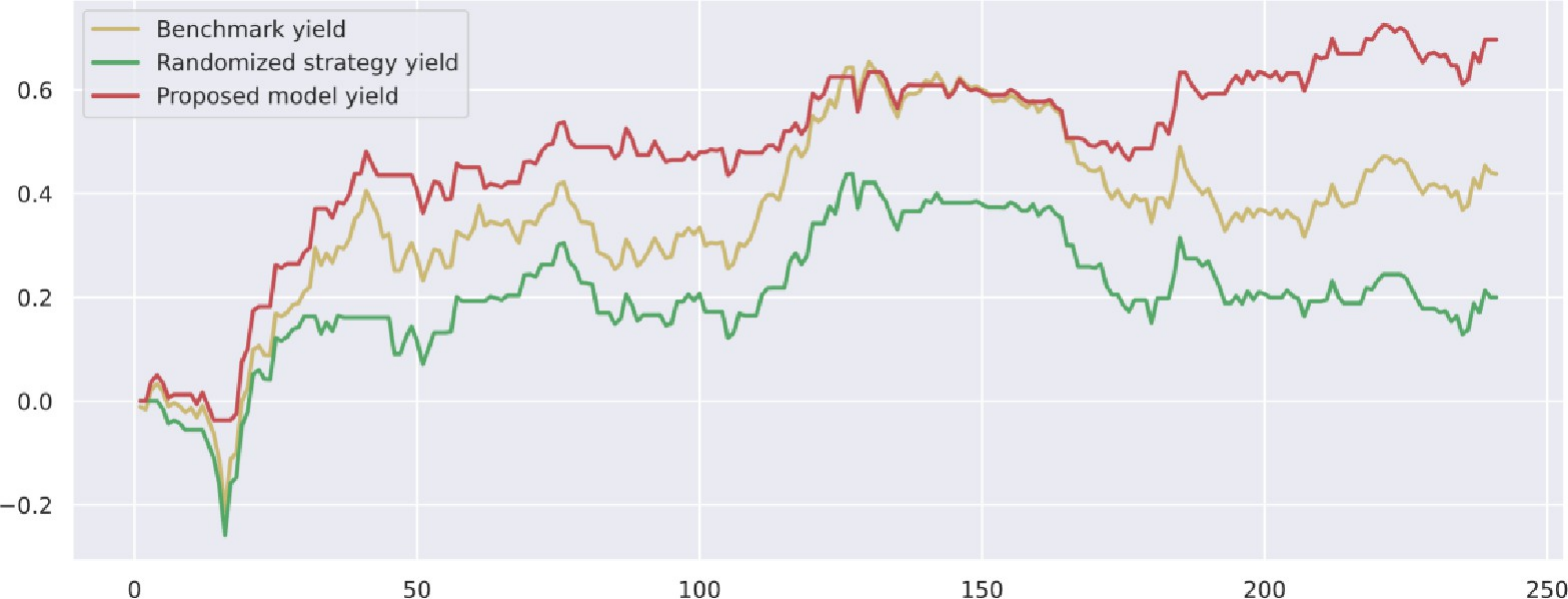
Mu yuan stock strategy returns.

**Fig 15:**
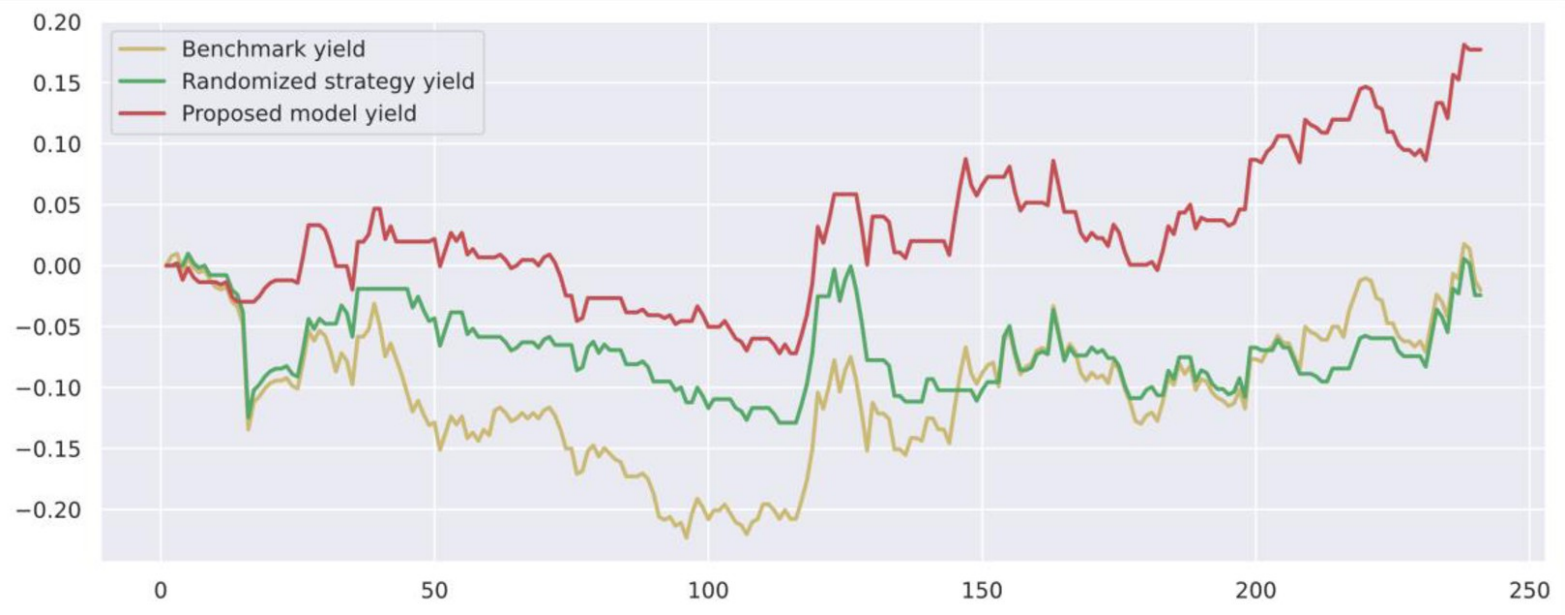
China nuclear power stock strategy returns.

**Fig 16:**
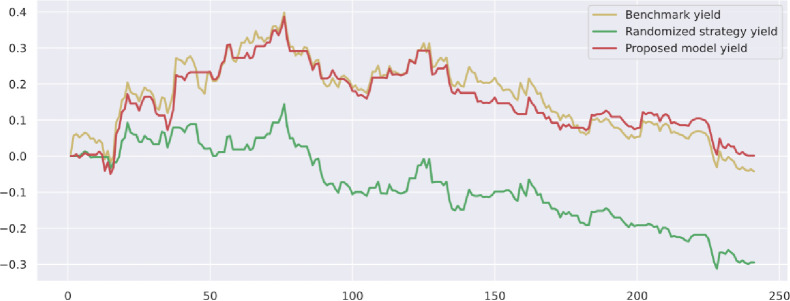
Yonghui supermarket stock strategy returns.

To demonstrate the value and practical effect of the proposed method for stock market risk warning and quantitative investment, we select the CSI 300 constituent stocks in the test set from 2020-01-01 to 2020-12-31 for backtesting. If the model predicts a rise, it generates a buy signal; a fall generates a sell signal: successive identical signals do not trigger trading operations. The initial capital of the strategy is 10,000, and the closing price of the trading day is used as the return settlement standard. The returns of the trading strategies constructed by the model are presented. Fig 10 shows the return on trading strategy investment and introduces the stochastic trading strategy and the benchmark returns for comparison. Figs 11–16 shows the returns of individual stocks extracted from the finance, public utilities, real estate, comprehensive, industrial and commercial sectors. The stochastic trading strategy is formed by randomly implementing 0 and 1 trading orders at the stock trading time points generated by the method in this paper.

Figs 10–16 and Table 6 present the returns of the trading strategy proposed in this paper. Due to the impact of the novel coronavirus epidemic in March 2020, the benchmark returned -14.37%, and the randomized strategy returned -14.21%. The investment return of the proposed method in this paper is 2.35%, which is the highest return and outperforms the stochastic trading strategy and the benchmark return. Since the shorting return in the stock market forecast is not considered in this paper’s strategy, the strategy return in this paper is closely related to the trend of the forecast index. The stock market experienced two sharp declines caused by sudden black swan events (novel coronavirus outbreak) with a maximum retardation of 27.17%, and the strategy developed in this paper played an important role in stopping losses due to better risk identification, resulting in better returns. Table 6 provides an overview of the strategy for quantitative investments. From the perspective of systematic risk, the alpha value is 0.36, the beta value is 1.312, and the systematic risk is greater than the nonsystematic risk. The Sharpe ratio is 0.95, and the Sortino ratio is 1.23, indicating that each downside risk can lead to a greater excess return. The average information ratio is 1.12, indicating that excess risk brings more excess return than does general risk. A smooth investment return was achieved under the impact of the novel coronavirus outbreak. Faced with a large amount of news data, the method proposed in this paper is accurate and rapid for stock market prediction. The method can not only account for sudden negative news but can also accurately extract effective information for predicting stock movements based on daily stock market news and fully explain the information spillover generated by the information correlation along the meta-path.

## 5. Summary and prospects

Based on the open world hypothesis, this paper constructs the complex network of stock market to realize multi-source heterogeneous data fusion. Node and semantic attention mechanisms are introduced to construct a graph neural network, and fully mine the node features and meta path semantic information of heterogeneous networks to complete the prediction of stock market price fluctuation. The multi-source heterogeneous information fusion and prediction method proposed in this paper is superior to the traditional method and extends the application of graph data and graph neural networks in the research of stock market price fluctuations. Although the method proposed in this paper improves the explanatory power of multi-source heterogeneous data information on stock market price fluctuations, there are many factors that affect stock market price fluctuations. We will try to expand the increase in the next step research trading data derivative index, graphical data, such as rich and complex network node types and features, further study of the interaction between the multi-source heterogeneous data information mechanism, and exploration to build a public opinion monitoring and information mining function of securities market risk prevention and control regulation system.

## Supporting information

S1 Data (DOCX)
