# Supplementary material for: A graph-based approach to multi-source heterogeneous information fusion in stock market
Source: PLoS One. 2022 Aug 11;17(8):e0272083. doi: 10.1371/journal.pone.0272083 (PMC9371341; doi:10.1371/journal.pone.0272083)
Supplement: S1 Data — (DOCX) [file pone.0272083.s001.docx]

1.The experimental data acquisition link is as follow: [https://figshare.com/s/7f924a6df9b4a345a3f0](https://figshare.com/s/7f924a6df9b4a345a3f0)

2.The sample code link: <https://figshare.com/s/2a001ca601b44ae0a4d0>
